# Evaluation of the Effects of Different Measurable Forces on the Viability of Bovine Nasal Septal Cartilage: In Vitro Experimental Study

**DOI:** 10.1007/s00266-026-05958-1

**Published:** 2026-06-10

**Authors:** Hatice Kadı Kardaş, Özgür Kemal, Abdurrahman Aksoy, Yavuz Kürşad Daş, Şerife Tütüncü, Şemsettin Kardaş, Onur Yontar, Özlem Terzi, Emel Tahir, Emre Demirel

**Affiliations:** 1 Department of Otolaryngology, Van Education and Research Hospital, Van, Turkey; 2 Department of Otolaryngology, Ondokuz Mayıs University School of Medicine, Samsun, Turkey; 3 Faculty of Veterinary Medicine, Department Pharmacology and Toxicology, Ondokuz Mayıs University, Samsun, Turkey; 4 Department of Histology and Embriyology, Faculty of Veterinary Medicine, Ondokuz Mayıs University, Samsun, Turkey; 5 Department of Mechanical Engineering, Faculty of Engineering, Ondokuz Mayıs University, Samsun, Turkey; 6 Department of Public Health, Ondokuz Mayıs University School of Medicine, Samsun, Turkey

**Keywords:** Rhinoplasty, Septorhinoplasty, Cartilage, Crushing, Graft survival, Cartilage viability

## Abstract

**Background:**

The viability of cartilage grafts crushed during septorhinoplasty procedures remains unpredictable, causing hesitation among surgeons. This study aimed to objectively evaluate how different measurable crushing forces influence the viability of bovine nasal septal cartilage, thus providing clearer guidance for clinical practice.

**Methods:**

A custom-designed tissue-crushing device incorporating a dynamometer was utilized to apply precise crushing forces of 200, 300, 400, and 600 Newtons (N) to bovine nasal septal cartilage samples. Following crushing, the samples underwent incubation in cell culture for four weeks. Cartilage viability was then assessed histopathologically and scored between 1 and 3 points. Statistical analysis compared viability scores across the different groups.

**Results:**

Significant differences were observed among the experimental groups. The control group demonstrated the highest viability (2.8 ± 0.42), followed by groups subjected to 200 N (2.6 ± 0.51), 300 N (2.2 ± 0.42), 400 N (1.8 ± 0.42), and 600 N (1.3 ± 0.48) crushing forces. Viability scores were significantly lower in groups exposed to forces of 400 N and 600 N (*p* < 0.001).

**Conclusion:**

The newly developed tissue-crushing device allows standardized, objective quantification of cartilage-crushing forces. Based on our findings, applying crushing forces of up to 300 N maintains acceptable cartilage viability, providing useful guidance for clinical applications and future research.

**Level of Evidence V:**

This journal requires that authors assign a level of evidence to each article. For a full description of these Evidence-Based Medicine ratings, please refer to the Table of Contents or the online Instructions to Authors www.springer.com/00266.

## Introduction

Grafts are frequently used in septorhinoplasty to obtain a structurally strong osseocartilaginous skeleton, to camouflage the sharp borders formed after hump resection and osteotomy, to fill potential cavities, and to obtain a smooth and soft nasal contour [1]. Although there are autografts, homografts and alloplastic materials that can be used for these purposes, autogenous cartilage tissue is highlighted as the ideal graft material due to its large, strong and easily shaped structure, biocompatibility, being generally available from the operation field and being easily fed [2]. Cartilage grafts can be used as blocks, cubes or crushed according to need. Some complications might occur if a surgeon uses cartilage grafts in rhinoplasty without any additional procedure. The visualization of cartilage grafts through the skin is the most important complication of using cartilage tissues in rhinoplasty directly as a dorsal onlay graft, or as a cap graft. To avoid this complication, the cartilage can be chopped or crushed before use [3]. As chopped cartilage grafts are easily dispersed under pressure in the area where they are placed, they may be used by wrapping them in the fascia to obtain a more voluminous tissue [4]. The need for another donor site for the temporal fascia and complications such as alopecia at the incision area are the most important disadvantages of chopped cartilage grafts. The unpredictability of the effects of cartilage crushing on the long-term viability of the graft causes surgeons to approach the use of crushed cartilage grafts with concern. Therefore, the aim of this study was to investigate the effects of crushing on cartilage viability and thereby contribute to reducing graft failure.

## Materials and Methods

**Fig. 1:**
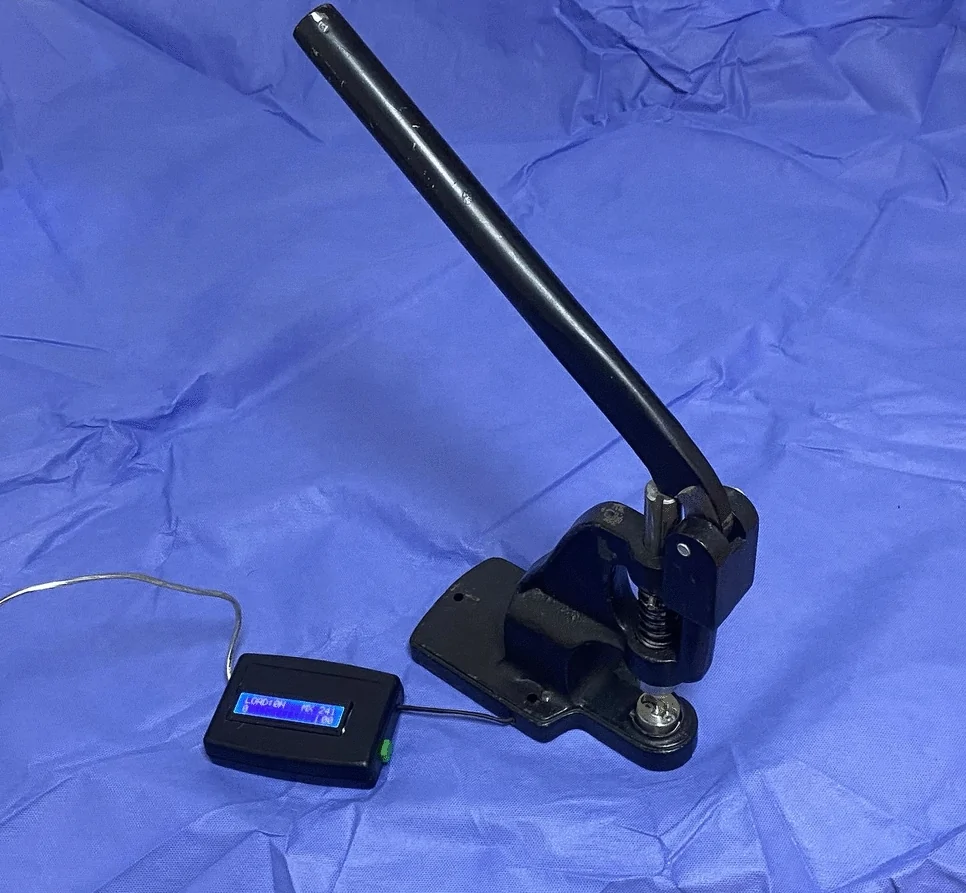
Tissue-crushing device with dynamometer

This in vitro investigation was conducted using animal tissue in the Veterinary Pharmacology and Toxicology laboratories of Ondokuz Mayis University. At the first stage of the research, a specialized apparatus was developed which incorporated a dynamometer to enable the precise quantification of the force exerted on tissues in units of Newtons (N) (Fig. 1).

Nasal septal cartilage was obtained postmortem from a healthy male Jersey bull, raised and slaughtered for routine meat production in a licensed abattoir under standard veterinary inspection. No procedures were performed on a living animal, and no animal was killed specifically for this research. Following slaughter, osteotomies were performed to obtain the nasal septum cartilage en bloc. Cartilage specimens (*n* = 50; 4 × 4 × 1 mm) were prepared consecutively from the central septum.

**Fig. 2:**
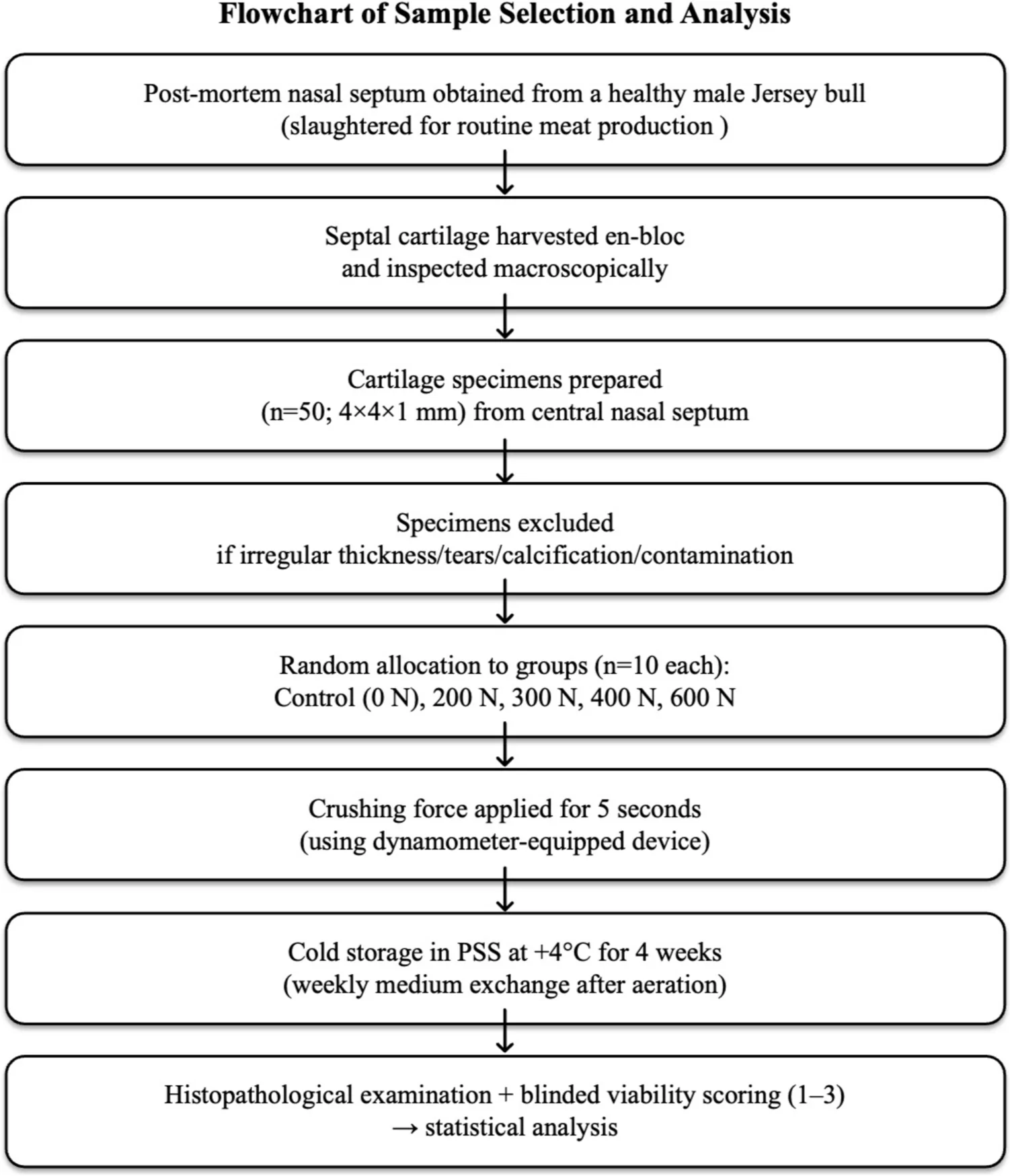
Flowchart of cartilage specimen selection, preparation, randomization, and histopathological evaluation

Septal cartilage segments with intact macroscopic structure and adequate thickness to prepare standardized specimens were included, and specimens with visible tears, marked irregular thickness, macroscopic contamination, calcification, or preparation-related defects were excluded. Specimens were numbered and allocated to study groups randomly to ensure equal group sizes and to minimize allocation bias. Figure 2 includes a flowchart of cartilage specimen selection, preparation, randomization, and histopathological evaluation.

**Fig. 3:**
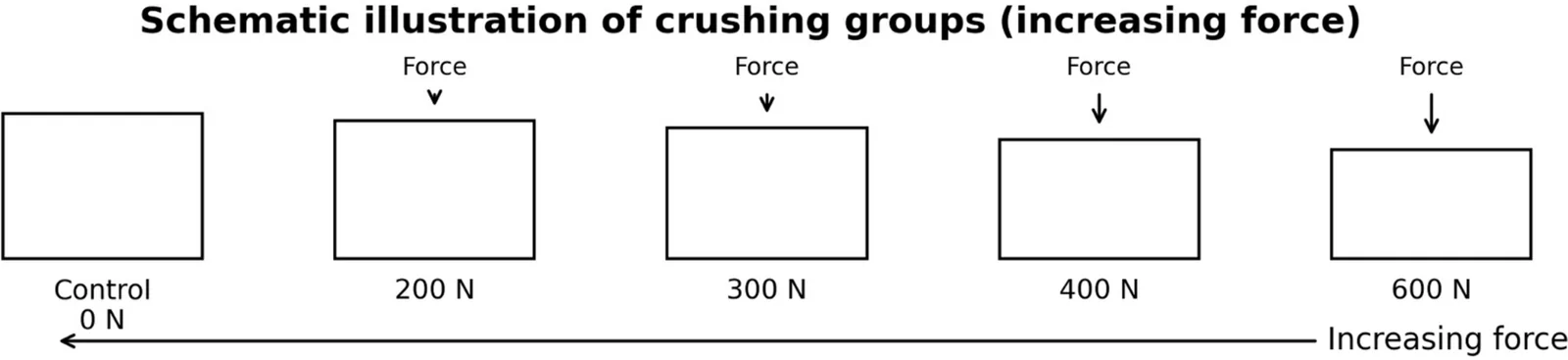
Simplified schematic illustrating the study groups and increasing crushing forces

Five separate groups were formed based on the magnitude of the exerted force, with each group comprising ten cartilage samples: Group 1 served as the control group, with no force applied, Group 2 samples were subjected to a force of 200 N, Group 3 to 300 N, Group 4 to 400 N, and Group 5 to 600 N. The forces were administered for a predefined duration of 5 seconds (Fig. 3).

Following the application of compressive forces, the specimens were subjected to 4 weeks of cartilage cultivation. During this phase, a physiological saline solution (PSS) was employed as the culture medium, formulated with the following constituents: sodium chloride (NaCl) at a concentration of 118 mM, potassium chloride (KCl) at 4.75 mM, calcium chloride dihydrate (CaCl_2_ × 2H_2_O) at 1.92 mM, sodium bicarbonate (NaHCO_3_) at 25 mM, potassium dihydrogen phosphate (KH_2_PO_4_) at 1.19 mM, magnesium sulfate heptahydrate (MgSO_4_ × 7H_2_O) at 1.19 mM, and dextrose anhydrous (dextrose X H_2_O) at 10.1 mM, all of which were procured from Merck, Germany. As a preemptive measure against microbial contamination, a concentrated solution of antibiotics and antimycotics was introduced into the PSS [5]. This solution comprised 10,000 units/mL of penicillin, 10,000 µg/mL of streptomycin, and 25 µg/mL of Gibco Amphotericin B from Thermo Fisher Scientific, USA.

**Fig. 4:**
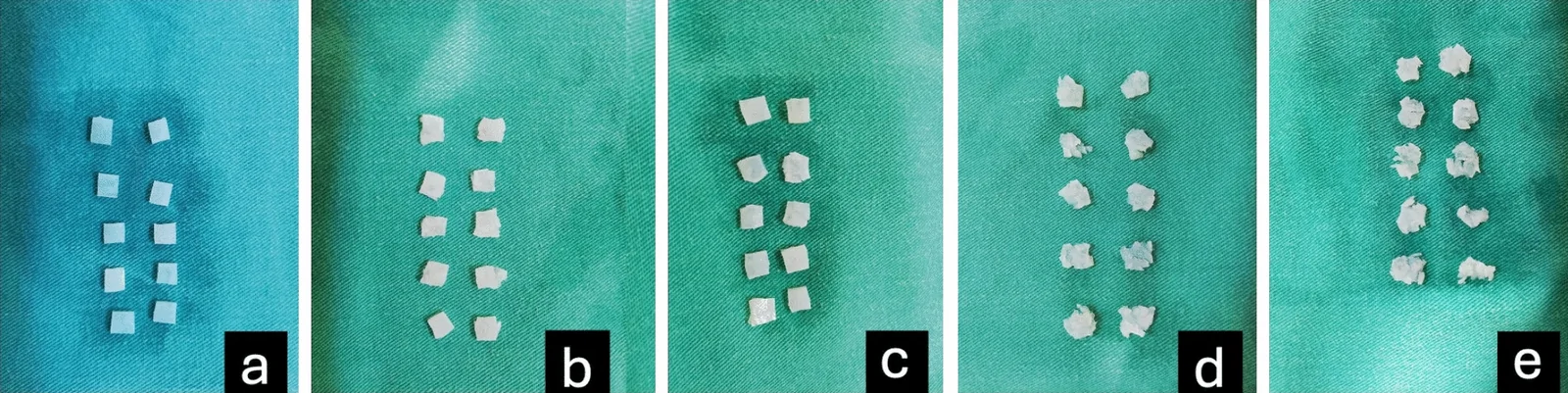
Intact and crushed cartilage samples after 4-week incubation in PSS at +4 °C; **a** control group (0 N), **b** 200 N (Group 2), **c** 300 N (Group 3), **d** 400 N (Group 4), **e** 600 N (Group 5)

Each experimental group was incubated within 50 mL of the prepared PSS, maintained at +4 °C, as a standardized cold-storage exposure condition applied identically across all groups. Prior to incubation, the PSS underwent aeration for 30 minutes. Subsequently, at weekly intervals, the PSS was replenished with freshly aerated solution. At the end of the 4-week period, all cartilage samples were removed from the culture media (Fig. 4) and underwent detailed histopathological analysis.

The histopathological evaluations were carried out by an experienced histologist. Following appropriate specimen acquisition techniques, the samples were fixed using a 10% formaldehyde solution. Standard protocols for histological tissue processing were then implemented, followed by embedding the specimens in paraffin blocks. Sections of 5 µm thickness were cut from the paraffin blocks. For the evaluation of chondrocyte viability and tissue integrity, both the Crossmon’s trichrome staining method and the hematoxylin–eosin staining method were used. The assessment of chondroid matrix metachromasia within the cartilage tissue was undertaken using the toluidine blue staining technique. The groups were compared using a comprehensive analysis of parameters including cartilage tissue cell characteristics, cellular viability, metachromasia of colloid tissue, and histopathological attributes [6].

Based on the detailed histopathological observations within each respective group, the extent of chondrocyte viability was graded on a scale ranging from 1 to 3 points. This assessment was performed by modifying the “cell population viability” item from the report prepared by the Histology Endpoint Committee of the International Cartilage Repair Society (ICRS) on histological assessment of cartilage repair. Predominantly viable group was assigned 3 points, intermediate viability 2 points, and partially viable 1 point [6]. The preparates obtained were photographed under a Nikon 50i research microscope, with a Nikon Digital Sight DS-Fi1 imaging system.

To assess intra-observer reliability, a subset of 10 specimens was randomly selected and re-evaluated by the same histologist after a washout period, blinded to initial scores. Intra-observer agreement was quantified using the intra-class correlation coefficient (ICC).

To determine the required sample size, a priori power calculation was performed using G*Power (version 3.1.7). Based on effect size estimates (*d* = 0.56) from a previous study [7] and assuming comparisons among five independent groups, a total sample size of 50 cartilage specimens (five groups of 10) was calculated to provide a power (1–β) of 0.90 at a two-sided α level of 0.05. Statistical analysis was performed using IBM SPSS Statistics for Windows, version 22.0 (IBM Corp., Armonk, NY, USA). The primary outcome (histological viability score) is ordinal; therefore, overall group differences were assessed using the Kruskal–Wallis test. When the omnibus test was significant, pairwise comparisons were performed using the Mann–Whitney *U* test. To control the familywise type I error rate for multiple pairwise comparisons, a Bonferroni adjustment was applied (adjusted *α* = 0.05/10 = 0.005 for 5 groups). As a sensitivity analysis, one-way ANOVA was additionally performed to evaluate concordance of conclusions across parametric and nonparametric approaches; omnibus effect size was reported as eta-squared (eta^2^). Figure 6 shows mean viability scores with 95% confidence interval error bars across groups.

## Results

### Histopathological Findings

Group 1 (Control Group): The cartilage tissue demonstrated a normative histological profile, characterized by the presence of regular structural attributes within the perichondrium, chondroblasts, and chondrocytes.

Group 2 (200 Newtons Pressure): Noteworthy in this group was the manifestation of mild tissue damage and localized degeneration within distinct regions of the cartilage tissue. Nevertheless, the salient features pertained to the well-preserved structural integrity of chondrocytes and the prominent presence of metachromasia, indicative of sustained cellular vitality.

Group 3 (300 Newtons Pressure): A subtle compromise in the overall tissue integrity within the cartilage matrix was observed. However, the histological composition and viability of chondroblasts and chondrocytes, in conjunction with the degree of colloid tissue metachromasia, were seen to be comparable with those of the initial control group.

Group 4 (400 Newtons Pressure): Significantly compromised tissue integrity was observed, surpassing the levels in Groups 2 and 3. Although the structural and viability attributes of chondroblasts and chondrocytes were discernible, there was a noticeable diminution in the metachromasia of the colloid tissue.

**Fig. 5:**
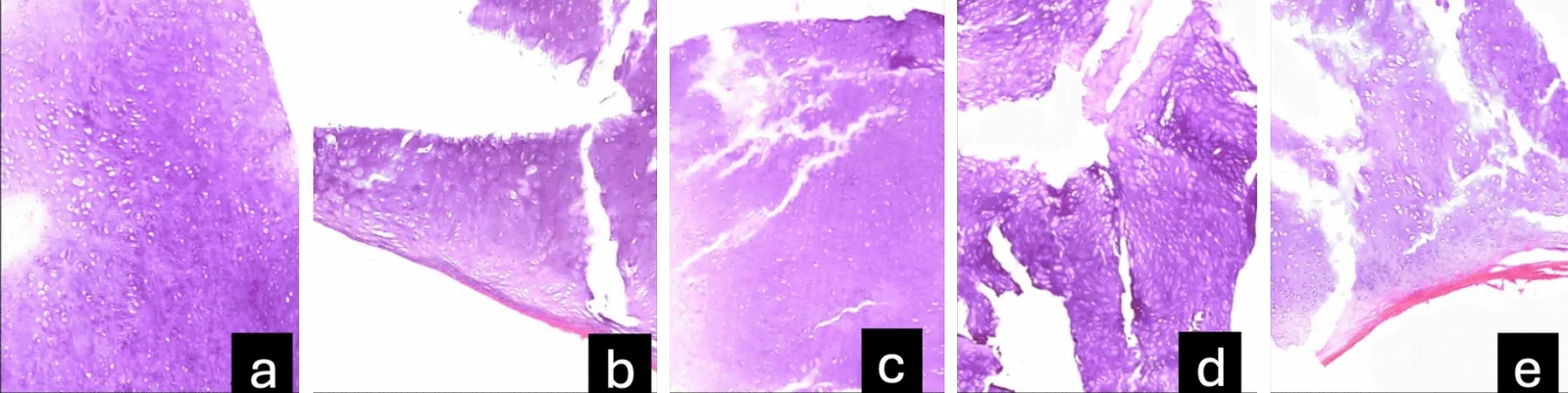
Representative histology (hematoxylin and eosin) at ×4 magnification; **a** intact cartilage tissue of Group 1, **b** cartilage tissue of Group 2, **c** cartilage tissue of Group 3, **d** cartilage tissue of Group 4, **e** cartilage tissue of Group 5

Group 5 (600 Newtons Pressure): Pronounced disruption of tissue integrity within the cartilage became evident. Compared with the other groups, particularly the control group (Group 1), this group exhibited markedly diminished cellular viability, intensified metachromasia, and exacerbated tissue damage (refer to Figure 5).

### Viability Scores

**Fig. 6:**
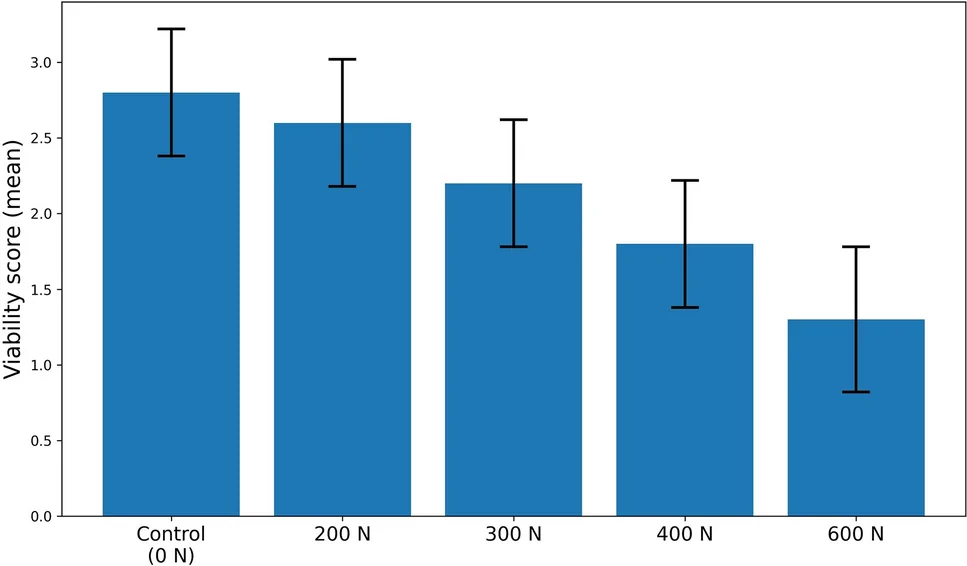
Mean viability scores with 95% confidence interval error bars across groups

Significant differences were observed among groups in the mean and median tissue viability scores. The mean viability scores were 2.8 ± 0.42 (95% CI 2.50–3.10) (control), 2.6 ± 0.51 (95% CI 2.24–2.96) (200 N), 2.2 ± 0.42 (95% CI 1.90–2.50) (300 N), 1.8 ± 0.42 (95% CI 1.50–2.10) (400 N), and 1.3 ± 0.48 (95% CI 0.96–1.64) (600 N) (Table 1) (Fig. 6). Intra-observer reliability was excellent, with an ICC of 0.93 (95% CI 0.74–1.00). As a sensitivity analysis, one-way ANOVA confirmed an overall difference among groups (*p* < 0.001; eta^2^ = 0.62).

**Table 1:** Mean and median viability scores of groups

	Mean±SD	Median (min–max)	^a^*p*
Group 1	2.8 ± 0.42 (2.50–3.10)	3(2–3)	*p* < 0.001
Group 2	2.6 ± 0.51 (2.24–2.96)	3(2–3)	
Group 3	2.2 ± 0.42 (1.90–2.50)	2(2–3)	
Group 4	1.8 ± 0.42 (1.50–2.10)	2(1–2)	
Group 5	1.3±0.48 (0.96–1.64)	1(1–2)	

*SD* standard deviation, *CI* confidence interval, min, minimum; max, maximum

^a^Kruskal–Wallis Test

The viability scores of the control group (Group 1) with no force applied were significantly higher than those of the cartilage groups subjected to 400 N (Group 4) and 600 N (Group 5) crushing forces (*p* = 0.001 and *p* < 0.001, respectively).

The viability scores of Group 2 were significantly higher compared to Group 4 and Group 5 (*p* = 0.003, *p* < 0.001, respectively). However, no significant difference was observed between Group 2 and Group 3 (*p* = 0.143).

The viability scores of the Group 3 cartilage samples were found to be significantly higher than those of Group 5 (*p* = 0.003), whereas no statistically significant difference was found between Groups 3 and 4 (*p* = 0.190) (Table 2).

**Table 2:** *P* values from Mann–Whitney *U* tests for pairwise comparisons of viability scores between groups

	Group 1	Group 2	Group 3	Group 4	Group 5
Group 1	–	0.481	0.023	0.001*	< 0.001*
Group 2	0.481	–	0.143	0.003*	< 0.001*
Group 3	0.023	0.143	–	0.190	0.003*
Group 4	0.001*	0.003*	0.190	–	0.063
Group 5	< 0.001*	< 0.001*	0.003*	0.063	–

^*^Significant after Bonferroni correction (*α* = 0.005)

## Discussion

In septorhinoplasty operations, crushed cartilage grafts are used as cap grafts for augmentation of the nasal tip, or as dorsal onlay grafts to provide an esthetically flatter and smoother dorsal appearance [3]. The inability to predict when and to what extent the crushing of the cartilage will affect the viability of the graft and the lack of a clear standardization of the crushing procedure cause surgeons to approach the use of these grafts with concern. There are few studies on the impact of cartilage crushing on cartilage viability, and the results of those studies are inconsistent. The survival rates of crushed cartilage grafts have been reported in literature to be between 10 and 90%, and it is not possible to reach a clear conclusion on this subject with the available data [7, 8].

Bujia and Verwoerd-Verhoef et al. reported that the viability of crushed cartilages is very low and they should not be used for mechanical support [7, 9]. However, Breadon et al., Guyuron and Friedman concluded that crushed cartilage can be used safely with similar success rates to those of uncrushed cartilage grafts [10, 11].

Attributing this inconsistency in previous study results to the non-standardization of the crushing process, Çakmak et al. performed the crushing process with the help of a Cottle cartilage crusher in their experimental studies and graded the crushing force as follows: slight crushing: 1 medium-strength strike that will soften the surface of the cartilage without impairing its elastic strength; moderate crushing: 2 medium-strength strikes to soften the surface and reduce elastic resistance; severe crushing: 3 or 4 moderate impacts that will cause the graft to bend under the effect of gravity; and significant crushing: 5–6 strong strikes that will destroy the integrity of the cartilage [12]. In the study conducted using this classification, Cakmak et al. reported the viability rates of intact, slightly, moderate, severely, and significantly crushed cartilage groups as 90%, 70%, 50%, 30%, and 10%, respectively, and stated that the findings did not show any significant change in the 10-month follow-up period [13]. Other studies that have used the crushing classification described by Cakmak et al. have reported that slight and moderate crushing does not have a significant negative effect on chondrocyte viability [14, 15].

Evaluation of the strength of the hammer hit and the elasticity of the cartilage is completely subjective, and Boccieri et al. and Kayabaşoğlu et al. attempted different methods. In an experimental study by Kayabaşoğlu et al., the crushing process was performed with a Cottle cartilage crusher and morselizer and the viability rates were compared [8]. In a clinical study, Boccieri et al. performed the crushing process with a Cottle cartilage crusher, and evaluations were made by palpation and photographing. The ideal crushing force was defined as moderate, with the graft being elastic and tending to return to its original position when compressed and released, and having a “crocodile skin” appearance under light [16].

When the studies in the literature are examined, it can be observed that the cartilage-crushing process is generally performed with a Cottle cartilage crusher. The crushing process with the Cottle cartilage crusher is based on the application of force by hitting the cartilage graft placed between the metal planes with a hammer. The degree of crushing was classified by Çakmak et al., and in the study by Boccieri et al. it was based on the evaluation of the loss of elasticity in the cartilage by subjective methods, and thus, it was attempted to standardize the ideal crushing level [13, 15]. However, the fact that the working principle of the crushing device used is not based on the application of a quantitative force on the cartilage does not make an objective standardization method possible. Based on this shortcoming in the literature, the current study was started by designing a tissue-crushing device with a dynamometer. Therefore, objective quantitative data could be obtained in this study, which was considered to be a step toward solving one of the biggest deficiencies in the literature on this subject. Bovine nasal septum cartilage was selected as the study material because it is technically easier.

In the current study, the cartilage specimens were monitored in vitro under standardized conditions (+4 °C PSS with weekly medium exchange), which enabled direct assessment of force-dependent histological viability without confounding host inflammatory responses. However, as an in vitro model, it does not capture inter-individual biological variability or host immune and reparative responses to crushed cartilage.

In parallel with the previous studies in the literature, a significant decrease in cartilage viability was observed in the current study as the crushing force applied on the cartilage increased [8, 13, 16]. The results show that for cartilage at the dimensions used in this study, crushing force of up to 300 Newtons can be used safely in septorhinoplasty, and that forces applied up to 300 Newtons will result in limited resorption, but the application of forces of 400 Newtons and above will not be suitable for the use of cartilage grafts.

The in vitro monitoring of the crushed cartilage tissues in the current study had some limitations. First, the long-duration (4-week) incubation of specimens in cold physiological saline at +4 °C may introduce cold-storage and nutrient-deprivation effects; therefore, the observed viability may reflect a combination of crushing-related injury and cold-storage-related injury, and absolute viability estimates may be conservative. Nevertheless, because all groups were subjected to the same storage protocol, the between-group differences remain informative regarding relative force-dependent effects within this experimental model. Second, instead of using a physiological in vitro culture medium (e.g., DMEM supplemented with fetal bovine serum at 37 °C), our use of PSS at +4 °C may underestimate the viable cell fraction. Also sex-related effects could not be assessed because all specimens came from a single male donor; future multi-donor investigations including both sexes might be needed. Finally, this is an in vitro model that does not incorporate host biological responses or inter-individual variability; the findings should be interpreted as mechanistic and hypothesis-generating. Future studies should repeat these experiments under more physiological culture conditions and in vivo models to better isolate crushing-related effects and improve clinical generalizability.

The histopathological data were similar to those of previous studies in the literature, indicating that mild and moderate crushing does not have a significant negative effect on cartilage viability [14, 16, 17]. Unlike earlier studies that could not determine objective parameters of crushing intensity, the current study objectively quantified mild, moderate, and severe crushing forces using a dynamometer-equipped device. Although this force-measuring system is a laboratory prototype, the quantitative data it provides can enable objective standardization of cartilage crushing in future research, facilitate comparison across studies, and guide the development of clinically usable cartilage-crushing instruments with force-feedback or force-limiting mechanisms. In addition, these measurements can be used to calibrate conventional cartilage crushers by mapping subjective descriptors (e.g., mild/moderate/severe crushing) to objective Newton force ranges. Thus, the optimum crushing level may be quantitatively determined and, in the future, translated into septorhinoplasty practice to improve the predictability of graft viability when crushed cartilage is used as camouflage or filling material and potentially reduce early revisions related to graft necrosis.

## Conclusion

Optimal cartilage crushing should balance flexibility with the preservation of tissue viability. Based on this study, an applied force of approximately 300 Newtons was determined as optimal for the cartilage size investigated. However, additional in vivo and clinical research with narrower force ranges and larger sample sizes is necessary to validate these findings further. The tissue-crushing device developed for this study offers an objective and standardized methodology, potentially reducing the incidence of revision surgery in septorhinoplasty by improving graft predictability and viability.
